# An Improved Global and Local Fusion Path-Planning Algorithm for Mobile Robots

**DOI:** 10.3390/s24247950

**Published:** 2024-12-12

**Authors:** Yongliang Shi, Shucheng Huang, Mingxing Li

**Affiliations:** 1 School of Computer Science, Jiangsu University of Science and Technology, Zhenjiang 212100, China; 221210701219@stu.just.edu.cn; 2 School of Electrical and Information Engineering, Jingjiang College, Jiangsu University, Zhenjiang 212013, China

**Keywords:** mobile robot, global path-planning algorithm, local path-planning algorithm, fusion path-planning algorithm

## Abstract

Path planning is a core technology for mobile robots. However, existing state-of-the-art methods suffer from issues such as excessive path redundancy, too many turning points, and poor environmental adaptability. To address these challenges, this paper proposes a novel global and local fusion path-planning algorithm. For global path planning, we reduce path redundancy and excessive turning points by designing a new heuristic function and constructing an improved path generation method. For local path planning, we propose an environment-aware dynamic parameter adjustment strategy, incorporating deviation and avoidance dynamic obstacle evaluation factors, thus addressing issues of local optima and timely avoidance of dynamic obstacles. Finally, we fuse those global and local path-planning improvements to form our fusion path-planning algorithm, which can enhance the robot’s adaptability to complex scenarios while reducing path redundancy and turning points. Simulation experiments demonstrate that the improved fusion path-planning algorithm not only effectively addresses existing issues but also operates with higher efficiency.

## 1. Introduction

In recent years, mobile robots have been widely used in various applications due to rapid technological advancements [1], such as robot vacuums and automated guided vehicles. In the field of mobile robots, path planning plays a fundamental role; it facilitates efficient and reliable navigation of robots to their intended destinations by determining optimal routes from the starting to the ending points or routes that meet specific conditions [2]. With the active involvement of numerous scholars in research, path-planning algorithms have made significant progress. Building upon existing algorithms, mobile robots can autonomously plan and navigate in static environments. However, during navigation, they often generate excessive redundant paths and turning points, leading to unnecessary time and energy consumption. Worse, these algorithms present poor adaptability in dynamic environments with unexpected obstacles, resulting in issues such as getting stuck in dead ends or colliding with dynamic obstacles. Therefore, reducing path redundancy and turning points and enhancing adaptability in complex environments remain crucial challenges in path-planning algorithms.

Based on the applicable scenes of path planning, existing algorithms are mainly divided into two categories: global and local path-planning algorithms. Global path-planning algorithms are designed to plan a feasible path for mobile robots from their current position to their destination in a known environment. This category of algorithms includes the RRT (rapidly exploring random tree), ant colony algorithm [3], bee colony algorithm [4], particle swarm optimization [5], Dijkstra algorithm [6], and A* algorithm [7]. Local path-planning algorithms primarily determine the movement direction and speed of the device based on the local environment around the mobile robot, guiding the device to move while preventing collisions with surrounding obstacles; this category mainly includes the dynamic window approach (DWA) [8], time elastic band (TEB) [9], and artificial potential field (APF) [10]. With the assistance of certain control theories, these algorithms can help mobile robots perform specific operations; the primary control theories include techniques such as model predictive control (MPC) [11], proportional-integral-derivative (PID) control [12], and quantized fuzzy feedback control [13], among others.

Both types of algorithms have certain limitations. Global path-planning algorithms cannot assist robots in moving, and they perform poorly in dynamically changing environments. Local path-planning algorithms are prone to getting stuck in local optima in environments with complex obstacle distributions. Therefore, as the demand for intelligent path planning increases, combining these two types of algorithms has become a key research focus. The main steps of the fusion process are as follows: first, obtain a global map of the environment surrounding the mobile robot; next, use a global path-planning algorithm to get a path from the robot’s current position to the destination; finally, employ a local path-planning algorithm to continuously guide the mobile robot along the planned global path, ultimately helping it reach its destination. Although fusion algorithms partially address the issue of poor path quality, they often suffer from the suboptimal performance of the combined global and local path-planning algorithms. This results in unnecessary, redundant paths during robot navigation and poor adaptability in complex environments.

To overcome the issues of the above algorithms, this paper proposes a novel fusion path-planning algorithm. It can effectively reduce path redundancy and enhance the adaptability of mobile robots in complex environments. The main contributions are summarized as follows:

(1) For global path planning, we design an improved heuristic function to optimize the path search direction. Additionally, we adopt measures such as removing redundant paths and utilizing binary search, which effectively address issues of path redundancy and excessive turning points. Through comparative experiments, it has been demonstrated that the improved path-planning algorithm proposed in this study has relatively lower time consumption compared to other algorithms, such as A*, JPS, and sampling-based algorithms. Additionally, the path length and the number of turns have been significantly reduced, contributing to faster path planning for mobile robots.
(2) For local path planning, we introduce an environment-aware dynamic parameter adjustment strategy and construct evaluation factors for deviation and avoidance of dynamic obstacles. This resolves issues where existing algorithms easily fall into local optima and fail to avoid dynamic obstacles in a timely manner. Through comparative experiments, it has been demonstrated that the improved local path-planning algorithm is more effective at closely following the originally planned global path, and it also exhibits better performance in avoiding dynamic obstacles when encountered, offering greater stability and safety compared to other similar algorithms.
(3) We fuse the two novel strategies to form the fusion path-planning algorithm, addressing the problems of existing methods and enhancing the adaptability of mobile robots to complex scenes. By conducting simulation experiments in various scenarios, we demonstrate that compared to existing typical algorithms, our algorithm effectively solves current issues, offering advantages such as reduced path redundancy, fewer turning points, higher efficiency, and stronger adaptability.

## 2. Related Works

This section introduces existing path-planning algorithms, including global and local planning algorithms, as well as fusion algorithms that combine the search strategies of both.

### 2.1. Global Path-Planning Algorithms

Current global path-planning algorithms, based on their search methods, mainly include sampling-based algorithms, intelligent algorithms simulating biological behaviors, and graph search-based algorithms. Sampling-based algorithms primarily include RRT-class (rapidly exploring random tree) algorithms, such as RRT [14], RRT* [15], and informed-RRT* [16]. These algorithms employ a strategy of randomly generating routes, starting from the initial point and iteratively probing until the path search is complete. The paths generated by these algorithms often have numerous turns. Liao et al. [17] reduced the number and complexity of path turns by improving the selection mechanism of parent nodes and reducing redundant intermediate nodes. However, these algorithms still require continuous iteration for path optimization and struggle to navigate through narrow areas. Intelligent algorithms simulating biological behaviors mainly include the ant colony algorithm, bee colony algorithm, and particle swarm algorithm. These algorithms utilize iterative learning to complete path planning. Yu et al. [18] introduced the simulated annealing strategy into the particle swarm algorithm, accelerating its convergence speed. Wu et al. [19] incorporated a directional heuristic mechanism and an uneven initial pheromone distribution strategy into the ant colony algorithm, reducing its path complexity and search time. However, these algorithms demand high computational performance and have poor real-time capabilities. Graph search-based algorithms mainly include the Dijkstra algorithm and the A* algorithm. Graph search algorithms can quickly determine the feasibility of a path within a short time and explore a relatively short path, making them relatively suitable for mobile robots.

The A* algorithm is one of the most commonly used global path-planning algorithms. It utilizes a heuristic function strategy, allowing the search area to gradually approach the endpoint, ultimately finding the shortest path. However, as the map size increases, the search time significantly rises, and the generated path tends to have numerous turns, resulting in low algorithm efficiency and poor path quality. Li et al. [20] introduced a bidirectional search strategy in the A* algorithm search process, effectively reducing the search time but also increasing the path length. In 2011, Harbor et al. [21] proposed the JPS (jump point search) algorithm, which stores only necessary nodes during the search process, optimizing the search flow and greatly improving search efficiency while reducing the number of path points. However, this method can still generate many redundant paths. Zhang et al. [22] further optimized the heuristic function calculation in the JPS algorithm, giving it directionality to speed up the path search. They also reduced redundant path points and turns by detecting collisions, thereby further lowering path redundancy. However, the optimization effect of this method significantly decreases in areas with dense obstacles.

To address the shortcomings of existing global path-planning algorithms, this paper proposes an improved algorithm that effectively reduces path turns and redundancy across various scenarios.

### 2.2. Local Path-Planning Algorithms

Local path-planning algorithms consider the physical constraints of the mobile robot and its surrounding environment to regulate the robot’s range of motion speeds and enable autonomous obstacle avoidance, preventing anomalies during movement. These algorithms mainly include the time elastic band (TEB) algorithm, the artificial potential field algorithm, and the dynamic window approach (DWA) algorithm. The TEB algorithm balances path quality and time cost by adjusting the length of the path planning time and uses optimization algorithms to search for the optimal solution. However, in practical applications, it can encounter issues such as sudden changes in speed and the equipment being susceptible to impacts upon startup. The artificial potential field method guides the mobile robot by adding repulsive fields around obstacles and attractive fields around the target point, but this method can easily cause the robot to get stuck in a local optimum due to the balance of attractive and repulsive forces.

The dynamic window approach (DWA) algorithm is currently the most commonly used local path-planning algorithm. Proposed by FOX D et al., it is a highly practical dynamic path-planning algorithm that uses an evaluation function to help the device avoid obstacles and move, thereby completing navigation. However, its adaptability to dynamic environments is relatively poor. Marija et al. [23] enhanced the DWA algorithm by considering the presence of dynamic obstacles, transforming the movement of dynamic obstacles into changes in the occupied areas of the grid map, and incorporating the FD* (Focused D*) algorithm. This improvement gave the DWA algorithm some adaptability to dynamic environments. Wang et al. [24] introduced an obstacle density evaluation term into the DWA algorithm, reducing redundant paths and maximizing the distance from obstacles. However, in most experimental scenes, this led to a significant decrease in the device’s speed, resulting in reduced navigation efficiency.

To address the shortcomings of existing local path-planning algorithms, this paper proposes an improved algorithm that achieves better adaptability and navigation efficiency in various scenarios.

### 2.3. Fusion Path-Planning Algorithms

The fusion path-planning algorithm simultaneously employs the search strategies of the aforementioned two algorithms, enabling the mobile robot to achieve better adaptability across various scenes. Wu et al. [25] fused the smooth A* algorithm with the DWA algorithm, allowing the device to navigate more smoothly and closely along the global path. Li et al. [26] combined an improved A* algorithm with an improved DWA algorithm and applied it to agricultural robots, separating the obstacle avoidance weights for known and unknown obstacles in the DWA to flexibly adjust the device’s obstacle avoidance sensitivity. Sun et al. [27] fused the TEB algorithm with the A*-based improved Theta* algorithm, classifying obstacles into complete and incomplete obstacles, and used a hierarchical optimization method to reduce the complexity of path planning and improve path smoothness. Although these algorithms address the issue of poor path quality in fusion algorithms to some extent, they also significantly increase the computational overhead for mobile robots, reducing real-time performance during navigation.

Based on these facts, this paper proposes an improved fusion path-planning algorithm, which ensures good path quality and ease of navigation while maintaining better real-time performance and adaptability to complex environments.

## 3. Materials and Methods

The improved fusion path-planning algorithm is primarily divided into three parts: global and local path-planning algorithms, as well as algorithm fusion. Improvement strategies are proposed to address the issues in existing global and local path-planning algorithms, overcoming their deficiencies. The two improved algorithms are then fused using specific strategies, ultimately forming the improved fusion path-planning algorithm. The following sections describe the improvement strategies for both types of algorithms and the algorithm fusion strategy.

### 3.1. Improved Strategies for the Global Path-Planning Algorithm

Existing global path-planning algorithms mainly use heuristic functions and jump point search strategies to improve the speed of path search and planning. This paper focuses on improving the shortcomings of heuristic functions and path-generation methods.

#### 3.1.1. Optimization of the Heuristic Function

The heuristic function is used to determine the processing priority of a node; the smaller the heuristic function value of a node, the higher its priority for processing. The primary application of heuristic functions is in grid maps. After a mobile robot obtains environmental map data, it divides the data into cells of equal size, constructing a two-dimensional grid map.

**Figure 1:**
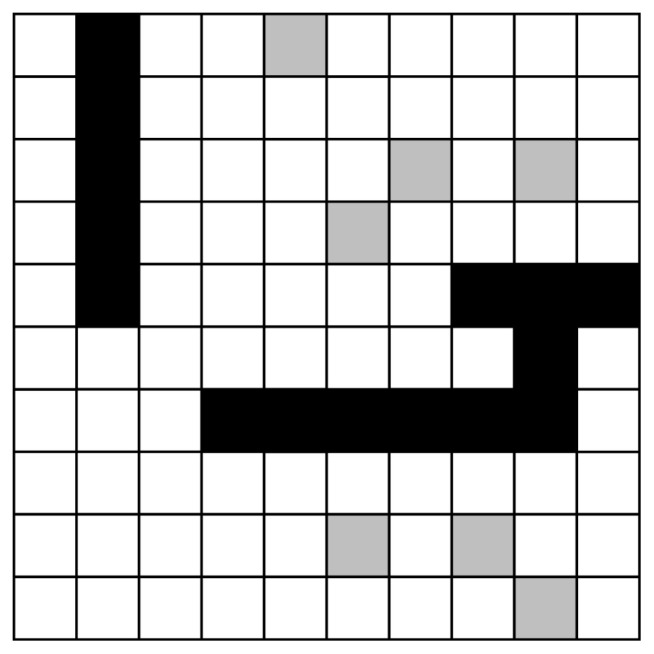
Grid map: White cells represent free space without obstacles, black cells represent occupied space with obstacles, and gray cells represent unknown space.

Each cell in the grid map can be referred to as a node. Each node has specific state information and coordinate information. As shown in Figure 1, the state information is mainly divided into three types: free space (white), occupied space (black), and unknown space (gray). The unknown space indicates that the region may contain obstacles previously undetected by the device.

**Figure 2:**
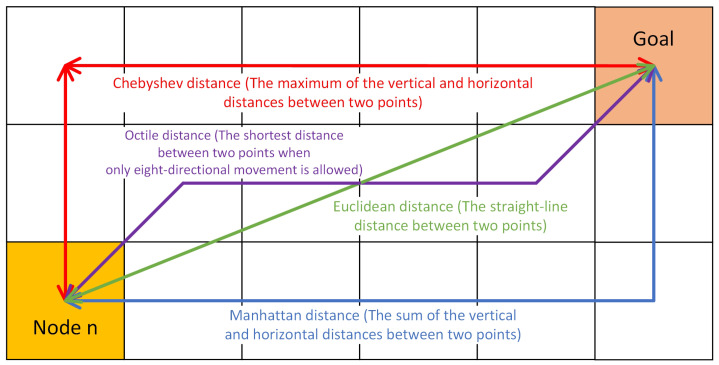
Diagram of various estimated costs. The estimated cost from node n to the goal can be measured in several ways: Euclidean distance (green) represents the straight-line distance between two points in two dimensions, the Manhattan distance (blue) is the sum of the horizontal and vertical distances between two points, the Chebyshev distance (red) is the maximum of the horizontal and vertical distances between two points, and the Octile distance (purple) is the shortest distance between two points when only eight-directional searches are allowed.

In a grid map, for a node n with coordinates $\left(x_{n},y_{n}\right)$, its heuristic function is defined as follows:

(1) $$f\left(n\right)=g\left(n\right)+h\left(n\right),$$

where $g\left(n\right)$ is the cost of moving from the starting point to node *n*. If the node is the starting point, then g(n)=0. Otherwise, let the parent node of *n* be *p*; then,

(2) $$g\left(n\right)=g\left(p\right)+\sqrt{{\left(x_{p}-x_{n}\right)}^{2}+{\left(y_{p}-y_{n}\right)}^{2}}.$$

h(n) is the estimated cost from node n to the goal. A good estimation method can significantly improve the algorithm’s efficiency. Commonly used estimation methods include Euclidean distance, Manhattan distance, Chebyshev distance, and Octile distance, as shown in Figure 2.

The existing heuristic functions have some shortcomings. The Octile distance considers only eight directions for searching, rather than any direction search. The Euclidean distance and Chebyshev distance may underestimate the cost in environments obstructed by obstacles, while the Manhattan distance may overestimate the cost, potentially leading to suboptimal node selection. Therefore, to balance speed and accuracy, this paper will adopt the following calculation method for estimating costs:

(3) $$\left\{\begin{array}{c} h\left(n\right)=\left(1-\varepsilon \right)L_{1}+\varepsilon L_{2}\\ \varepsilon =\left\{\begin{array}{c} \frac{d}{D}\;\;\;\;if\;\;d<D\\ 1\;\;\;\;\;\;\;\;otherwise \end{array}\right. \end{array},\right.$$

(4) $$L_{1}=\sqrt{(x_{n}-x_{goal}{)}^{2}+(y_{n}-y_{goal}{)}^{2}},$$

(5) $$L_{2}=\max\left(\left|x_{n}-x_{goal}\right|,\left|y_{n}-y_{goal}\right|\right)+\left(\sqrt{2}-1\right)min\left(\left|x_{n}-x_{goal}\right|,\left|y_{n}-y_{goal}\right|\right),$$

where $L_{1}$ and $L_{2}$, respectively, represent the Euclidean distance and Octile distance, *d* represents the straight-line distance from the current node to the endpoint, and *D* represents the straight-line distance from the starting point to the endpoint.

When the endpoint is farther away, with more obstacles obstructing the path, the weight of Euclidean distance decreases. When the endpoint is closer, with fewer obstacles, the weight of Euclidean distance increases. This improved heuristic function adapts to some extent to the global path search method, reduces path redundancy, and makes it more suitable for the global path search process.

#### 3.1.2. Optimization Strategy of the Path Generation

The path generation steps of the existing global path-planning algorithms  are shown in Algorithm 1.
**Algorithm 1** The path generation steps**Input:** $P_{start}$, $P_{goal}$, Map**Output:** Path *G*  1:Initialize Openlist and Closelist queues  2:Add $P_{start}$ to Openlist  3:**while** Openlist is not empty **do**  4:   Select the node *N* with the lowest heuristic function value from Openlist  5:   $P_{current}=N$  6:   Move *N* from Openlist to Closelist  7:   **if** $P_{current}$ == $P_{goal}$ **then**  8:     Tracing back to ancestral nodes  9:     Generate the path *G*10:     **break**;11:   **else if** $P_{current}$ == $P_{start}$ **then**12:     Search in all 8 directions13:   **else**14:     Search $P_{current}$’s parent node $P_{parent}$15:     **if** $P_{parent}$ is in a diagonal direction **then**16:        Search in the direction of the extended ray from $P_{parent}$ to $P_{current}$17:        Search the horizontal/vertical components of above direction18:     **else**19:        Search in the direction of the extended ray from $P_{parent}$ to $P_{current}$20:        Search the diagonal direction corresponding to any forced neighbors21:     **end if**22:   **end if**23:**end while**24:**if** *G* is not generated **then**25:   Path generation failed26:**else**27:   **return** *G*28:**end if**

If a jump point, obstacle, or boundary is encountered in any direction, the search in that direction is complete; when a jump point α is found in the search, the processing method is  shown in Algorithm 2.
**Algorithm 2** Handling of the jump point**Input:** Openlist, Closelist, α, $P_{current}$**Output:** Openlist, Closelist  1:**if** α∈Closelist **then**  2:   **break**;  3:**else**  4:   Calculate α’s new heuristic function value $f_{new}$  5:   **if** α∈Openlist **then**  6:     **if** α’s old heuristic function value $f_{old}>f_{new}$ **then**  7:        Update parent node of α to $P_{current}$  8:        Update heuristic function value of α to $f_{new}$.  9:     **end if**10:   **else**11:     Add α to Openlist12:     Set the parent node of α to $P_{current}$13:   **end if**14:**end if**

In the generation steps, a jump point is a node that satisfies specific conditions and is retained during the search process. When generating the final route, jump points are used to create the path. A node can be considered a jump point if it is the start or end point, if it has forced neighbors, or if its parent node is in a diagonal direction and there is a node in the horizontal or vertical component direction of the diagonal from the parent node to the current node that satisfies the two previously described conditions.

**Figure 3:**
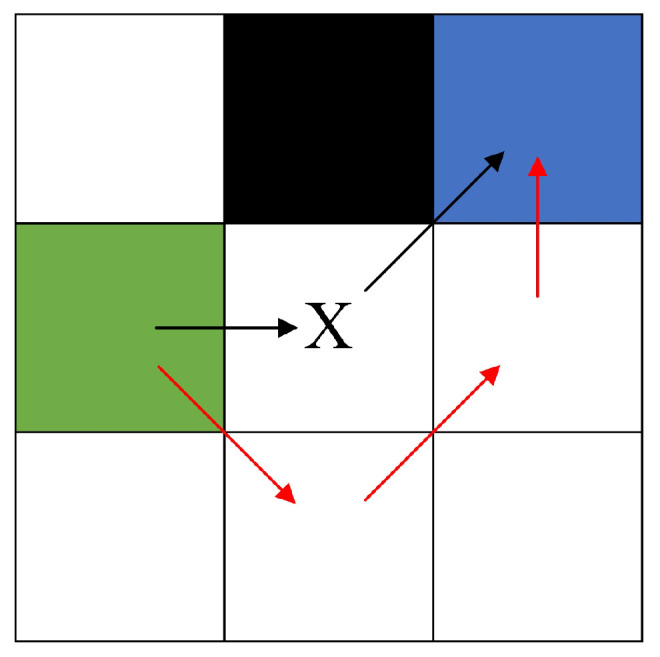
Diagram of forced neighbors. The black cell represents occupied space. The green cell represents *x*’s parent node. There are two ways to reach the blue cell *n* from the green parent node: one route passes through *x* along the black path with a shortest distance cost of $1+\sqrt{2}$, while the other route avoids *x* along the red path with a shortest distance cost of $1+2\sqrt{2}$. The former cost is less than the latter; thus, the blue cell is a forced neighbor of *x*.

In the context of jump point determination, a forced neighbor is defined as follows, as illustrated in Figure 3: node *n* is a traversable node in the eight squares surrounding node *x*; if there is an obstacle in the eight squares around traversable node *x*, the shortest path from *x*’s parent node to *n* must pass through *x*, and *n* is not a natural neighbor of *x* when there are no obstacles around *x*, then *n* is considered a forced neighbor of *x*.

**Figure 4:**
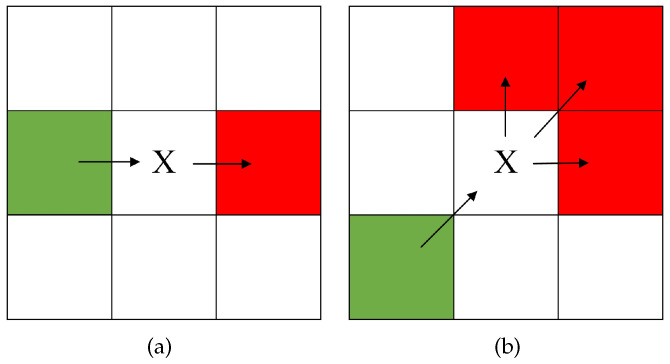
Diagram of natural nodes. Green cells represent *x*’s parent node, and red cells represent *x*’s natural node. (**a**) Parent node in *x*’s horizontal or vertical direction. (**b**) Parent node in *x*’s diagonal direction.

For natural neighbors used in forced neighbor determination, as shown in Figure 4, when there are no obstacles in the eight squares surrounding node *x*, *x*’s parent node may be either horizontally or vertically aligned with *x*, or it may be diagonally aligned. Depending on these two different scenarios, the positions of the natural neighbors will vary.

Existing global path-planning algorithms typically use eight-directional searches in grid maps, which can result in unnecessary path nodes and redundant paths. Therefore, it is necessary to optimize the path. The optimization strategy  is shown in Algorithm 3.
**Algorithm 3** Path optimization**Input:** Path *G*, map**Output:** The optimized path G’  1:Let the path nodes from the starting point to the endpoint be ($X_{0}$,$X_{1}$,$X_{2}$,…,$X_{n}$)  2:**for** i=n:1 **do**  3:   k = 0  4:   **for** j=n−1:0 **do**  5:     **if** $collsion(X_{i},X_{j})$== −1 **then**  6:        k = j + 1  7:        **break**;  8:     **end if**  9:   **end for**10:   **if** k==0 **then**11:     Update the parent node of $X_{i}$ to $X_{0}$12:     Update the path information to G’13:     **break**;14:   **end if**15:   $A=X_{k};B=X_{k-1};x_{C_{1}}=x_{A}+\frac{x_{B}-x_{A}}{2};y_{C_{1}}=y_{A}+\frac{y_{B}-y_{A}}{2}$16:   $F_{1}=collsion\left(X_{i},C_{1}\right)$17:   **for** l=2:m **do**18:     $x_{C_{n}}=x_{C_{n-1}}+F_{n-1}\cdot \frac{x_{B}-x_{A}}{2^{n}};y_{C_{n}}=y_{C_{n-1}}+F_{n-1}\cdot \frac{y_{B}-y_{A}}{2^{n}}$19:     $F_{l}=collsion\left(X_{i},C_{l}\right)$20:   **end for**21:   $x_{D_{1}}=x_{A}+\frac{x_{X_{n}}-x_{C}}{2};y_{D_{1}}=y_{A}+\frac{y_{X_{n}}-y_{C}}{2}$22:   $F_{1}=collsion\left(B,D_{1}\right)$23:   **for** l=2:m **do**24:     $x_{D_{n}}=x_{D_{n-1}}+F_{n-1}\cdot \frac{x_{X_{n}}-x_{C}}{2^{n}};y_{D_{n}}=y_{D_{n-1}}+F_{n-1}\cdot \frac{y_{X_{n}}-y_{C}}{2^{n}}$25:     $F_{l}=collsion\left(B,D_{l}\right)$26:   **end for**27:   $D=D_{m}$28:   Update the parent node of $X_{n}$ to *D*29:   Set the parent node of *D* to *B*30:   Update the path information to G’31:**end for**32:**return** $G^{\prime }$

**Figure 5:**
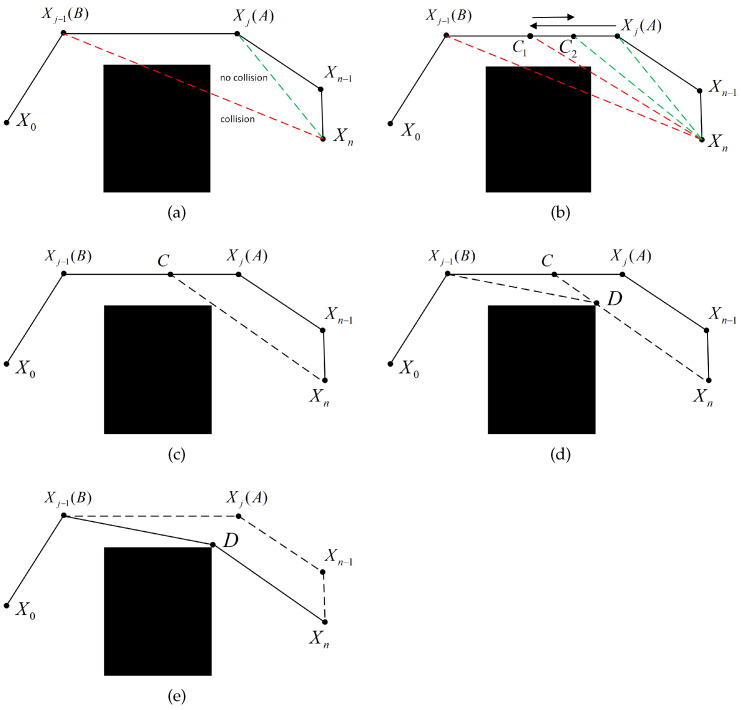
Globalpath generation optimization flowchart. (**a**) Backtrack to detect the ancestor node that collides. (**b**) Search for the critical point $C_{n}$ that avoids collision. (**c**) Obtain the critical point C that avoids collision. (**d**) Obtain the critical point D that avoids collision. (**e**) Complete the path optimization.

Where the function $collision(E_{1},E_{2})$ is used to determine whether the line segment $E_{1}E_{2}$ collides with the obstacle, if it collides, the value is −1. Otherwise, it is 1. *m* is the binary search frequency, which can be freely adjusted. The flowchart of path optimization is shown in Figure 5.

After completing the above operations, it should also start from the start point and change the search direction to search from the parent node to the optimal child node. Optimize the path by following the above algorithm until reaching the endpoint, ultimately achieving bidirectional path optimization.

### 3.2. Improved Strategies for the Local Path-Planning Algorithm

Existing local path-planning algorithms require establishing a kinematic model of the mobile robot. These algorithms use an evaluation function to score feasible speed samples and select the sample with the highest score as the driving strategy, ultimately completing the local path planning and guiding the robot to its destination. This paper addresses the deficiencies in the evaluation function of local path-planning algorithms by introducing a dynamic parameter adjustment strategy based on environmental perception. Additionally, it constructs new evaluation factors to further enhance the local path-planning algorithm.

#### 3.2.1. Kinematic Model and Evaluation Function

**Figure 6:**
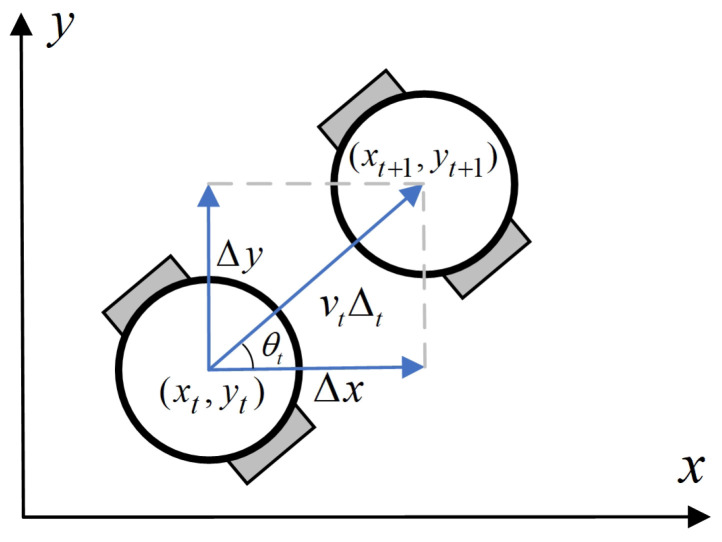
Kinematic model diagram of the robot, with positions before and after movement represented by $\left(x_{t},y_{t}\right)$ ans $\left(x_{t+1},y_{t+1}\right)$, respectively.

As shown in Figure 6, let the time increment from time *t* to t+1 be Δt. During this period, the mobile robot maintains motion. If Δt is short, the trajectory length of the robot from time *t* to t+1 can be considered as a short segment. At time *t*, if the robot moves with angular velocity $w_{t}$, linear velocity $v_{t}$, and the angle between the forward direction and the positive x-axis is $\theta_{t}$, then

(6) $$\left\{\begin{array}{c} \Delta x=v_{t}\Delta tcos\left(\theta_{t}\right)\\ \Delta y=v_{t}\Delta tsin\left(\theta_{t}\right) \end{array}.\right.$$

By accumulating the increments of displacement along the x-axis and y-axis over multiple time intervals, the position and heading direction of the mobile robot at any given time can be determined:

(7) $$\left\{\begin{array}{c} x_{t+1}=x_{t}+v_{t}\Delta tcos\left(\theta_{t}\right)\\ y_{t+1}=y_{t}+v_{t}\Delta tsin\left(\theta_{t}\right)\\ \theta_{t+1}=\theta_{t}+w_{t}\Delta t \end{array}.\right.$$

**Figure 7:**
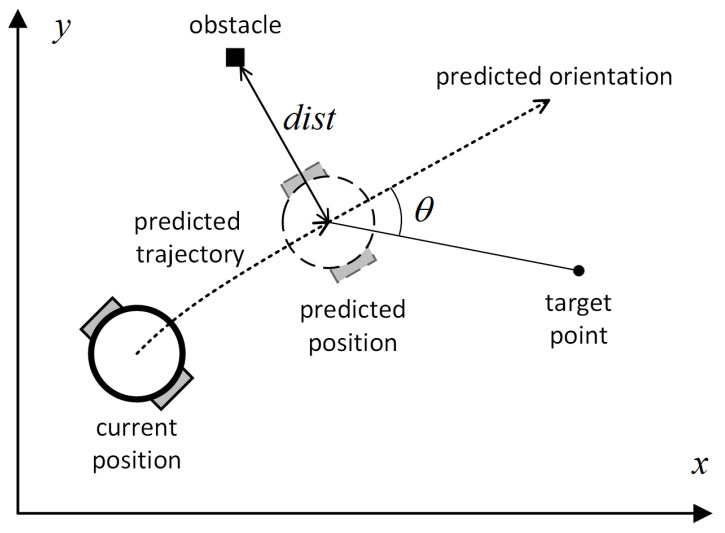
Diagram of the evaluation function, where θ is the angular difference between the robot’s orientation at the predicted endpoint and the target direction, and dist is the minimum distance between the robot’s position at the predicted endpoint and the obstacle.

On the basis of the kinematic model, existing local path-planning algorithms use evaluation functions to assess the trajectory corresponding to each feasibly sampled velocity space. The velocity space with the highest evaluation function value is then selected for actual path planning. The evaluation function is as follows:

(8) $$G\left(v,w\right)=\sigma \left(\alpha \cdot heading\left(v,w\right)+\beta \cdot dist\left(v,w\right)+\gamma \cdot velocity\left(v,w\right)\right),$$

where *v* and *w* represent the linear and angular velocities used by the mobile robot, respectively, $velocity\left(v,w\right)$ evaluates the magnitude of the current velocity space, with the score positively correlated with the speed. As shown in Figure 7, $heading\left(v,w\right)$ evaluates the angular difference θ between the robot’s orientation and the target direction when it reaches the predicted endpoint. The score is negatively correlated with the size of θ, and $dist\left(v,w\right)$ evaluates the distance between the robot and the nearest obstacle on this trajectory, with the score positively correlated with the distance.

The smoothing function σ(x) is used to normalize data to prevent any single factor from dominating the evaluation function. Let n denote the total number of valid trajectories, and i denote the corresponding trajectory number. The calculation method is as follows:

(9) $$\left\{\begin{array}{c} normal\_heading\left(i\right)=\frac{heading\left(i\right)}{\sum_{i=1}^{n}heading\left(i\right)}\\ normal\_dist\left(i\right)=\frac{dist\left(i\right)}{\sum_{i=1}^{n}dist\left(i\right)}\\ normal\_velocity\left(i\right)=\frac{velocity\left(i\right)}{\sum_{i=1}^{n}velocity\left(i\right)} \end{array}.\right.$$

#### 3.2.2. Dynamic Parameter Adjustment

In existing local path-planning algorithms, the weights of the evaluation function remain fixed during device operation, resulting in poor adaptability. By incorporating a dynamic weight adjustment mechanism that automatically changes according to the surrounding environment, the algorithm can become more adaptable. Among all the evaluation factors, the velocity(v,w) factor ensures that the robot travels at a relatively fast speed. However, in different environments, the weight allocation should vary. In obstacle-dense areas, the weight should be reduced to prevent collisions, while in less dense areas, the weight can be increased to enhance operational efficiency. Thus, the corresponding weight term is adjusted, as shown in Equation (10):

(10) $$\gamma^{\prime }=e^{k_{1}\cdot \left(dist\left(v,w\right)-d_{1}\right)},$$

where $k_{1}>0$ and $d_{1}$ represent the minimum safety distance between the mobile robot and obstacles. When the predicted trajectory is too close to obstacles, the weight of velocity magnitude is reduced; conversely, the weight is increased to enhance the device’s safety and flexibility.

#### 3.2.3. Deviation Evaluation Function

**Figure 8:**
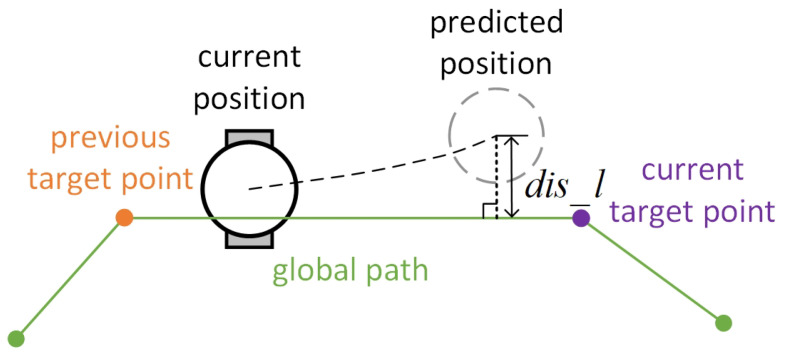
Diagram of deviation distance.

Existing local path-planning algorithms generally use a single path point from global path planning as the local target point. When unknown obstacles appear, the device temporarily deviates from the global path to avoid collisions. Frequent deviations may lead to additional unnecessary travel paths. To prevent excessive deviation from the path during the device’s operation, a new deviation evaluation factor p(v,w) is constructed, calculated as shown in Equation (11):

(11) $$p\left(v,w\right)=k_{2}\cdot \left(1-e^{-\frac{1}{\mu +dis\_l}}\right),$$

where μ>0, $k_{2}>0$, and dis_l represent the deviation distance from the position of the simulated trajectory endpoint in the corresponding velocity space to the straight line passing through the current target point and the previous target point. Introducing this scoring item encourages the device to actively return to the original global planned trajectory after deviating from the course, thereby enhancing path reliability, as shown in Figure 8.

#### 3.2.4. Dynamic Evasion Evaluation Function

**Figure 9:**
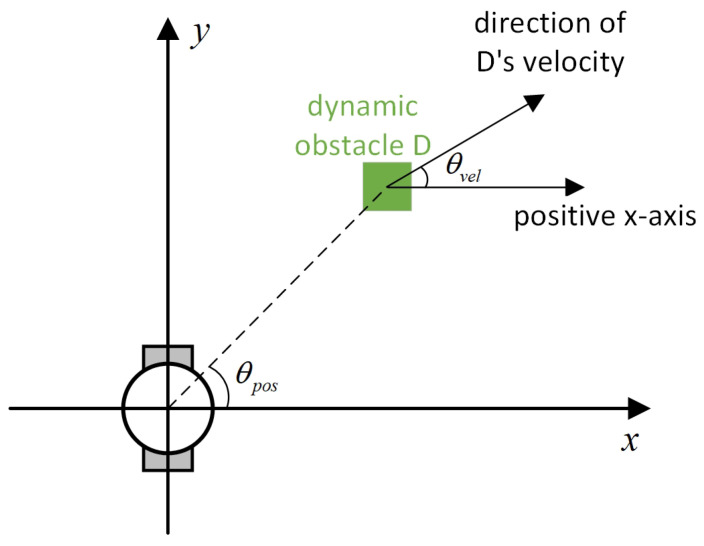
Diagram of $\theta_{pos}$ and $\theta_{vel}$, with the geometric center of the mobile robot set as the coordinate origin and the direction of the mobile robot’s velocity set as the positive x-axis.

Existing local path-planning algorithms often fail to avoid dynamic obstacles in a timely manner, leading to issues such as being too close to or even colliding with dynamic obstacles. To address these problems, an evaluation factor dyn(v,w) is constructed to avoid dynamic obstacles. The construction principle of this evaluation factor is as follows:

(1) Relative azimuth angle $\theta_{pos}$ and velocity angle $\theta_{vel}$
  As shown in Figure 9, the current velocity direction of the mobile robot is set as the positive x-axis of a newly established coordinate system, and the counter-clockwise 90° direction from the current velocity direction is set as the positive y-axis. The current position of the mobile robot is the origin of this new Cartesian coordinate system. Based on the position coordinates of the dynamic obstacle D, calculate its azimuth angle $\theta_{pos}$ relative to this new coordinate system, and also calculate $\theta_{vel}$, which represents the direction of D’s velocity in the newly established coordinate system.
(2) Collision risk assessment
  Based on the relationship between $\theta_{pos}$ and $\theta_{vel}$, different collision risk scenes arise, as shown in Table 1.
  Based on the classification from the table above, the collision risk level is divided into four levels from 0 to 3, ranging from low to high. Level 0 indicates no collision risk.
(3) Construction of the dyn(v,w) evaluation term
  The calculation method for the dyn(v,w) evaluation term is as follows:
  (12) $$dyn\left(v,w\right)=risk\cdot dist\_dy,$$
  where dist_dy represents the distance from the corresponding trajectory to the dynamic obstacle, and risk is the weight assigned based on the collision risk between the current moving robot and the moving obstacle, increasing with higher risk levels.
  The specific numerical values for risk are shown in Equation (13).
  (13) $$\left\{\begin{array}{c} 0\;\;\;\;\;\;\;\;\;\;\;\;\;\;\;\;\;\;\;d_{dyn}>d_{2}\\ level\;\;\;\;\;\;\;\;\;\;\;\;d_{3}<d_{dyn}\leq d_{2}\\ level+k_{3}\;\;\;\;\;\;0\leq d_{dyn}\leq d_{3} \end{array},\right.$$
  where $k_{3}>0$, $d_{dyn}$ represents the distance between the current moving robot and the dynamic obstacle, and $d_{2}$ denotes the maximum safety distance. $d_{3}$ represents the danger distance; when $d_{dyn}$ is less than the danger distance, it indicates a higher collision risk with the dynamic obstacle. Therefore, the collision level should be appropriately increased to ensure safety.

#### 3.2.5. Evaluation Function for the Improved Local Path-Planning Algorithm

Combining the previous improvements, the evaluation function of the improved local path-planning algorithm $G^{\prime }\left(v,w\right)$ is as shown in Equation (14):

(14) $$\left\{\begin{array}{c} g\left(v,w\right)=\alpha \cdot heading\left(v,w\right)+\beta \cdot dist\left(v,w\right)+\gamma^{\prime }\cdot velocity\left(v,w\right)\\ G^{\prime }\left(v,w\right)=\sigma \left(g\left(v,w\right)+\delta \cdot p\left(v,w\right)+\vartheta \cdot dyn\left(v,w\right)\right) \end{array}.\right.$$

### 3.3. Algorithm Fusion

The improved global and local path-planning algorithms have their respective limitations in different environments. Therefore, fusing the strategies of these two algorithms results in a more effective path-planning solution, leading to an improved fusion path-planning algorithm.

**Figure 10:**
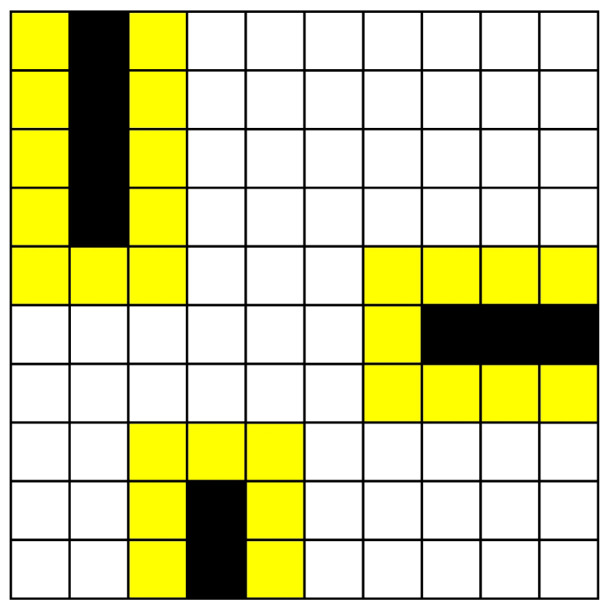
Diagram of inflation radius: in the diagram, the inflation radius is 1.5 units, and the inflated space is depicted in yellow cells.

In order to prevent the mobile robot from colliding with the surrounding environment due to its size, it is necessary to add an inflation radius when preprocessing the map. If the minimum Euclidean distance between a free space and an occupied space is smaller than the inflation radius, the free space is considered part of the occupied space. As shown in Figure 10, the yellow cells represent the inflated space. During global path planning, the inflated space is also treated as occupied space to reduce the risk of collisions during the robot’s movement. 

**Figure 11:**
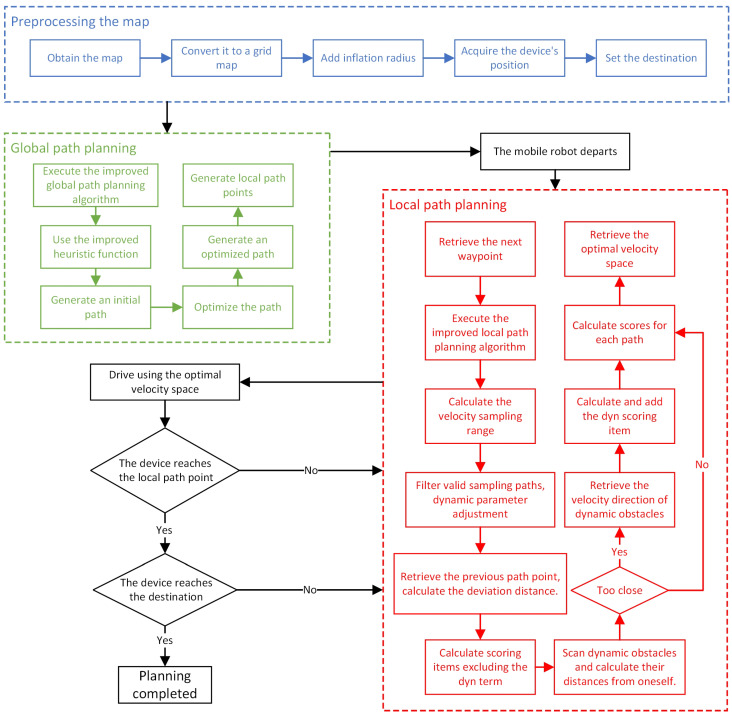
Flowchart of the improved fusion path-planning algorithm. After the mobile robot completes map acquisition and processing, it executes the improved global path-planning algorithm to generate local path points. The robot starts from the starting point, continuously treats the next local path point as the current target, and eventually reaches the endpoint.

The main process of the improved fusion path-planning algorithm is illustrated in Figure 11:

## 4. Experiments and Results

To verify the optimization effect of the improved algorithm, this paper conducts simulation experiments using MATLAB. The MATLAB version used is R2022b, and the operating system platform for the experiments is Windows 11, running on an i9-13900h processor with 16 GB of RAM.

### 4.1. Comparative Experimental Analysis of Global Path-Planning Algorithms

This subsection utilizes A*, JPS, the improved JPS algorithm from reference [22], and the improved global path-planning algorithm proposed in this paper. Additionally, it compares these methods with the sampling-based RRT* algorithm and F-RRT* algorithm from reference [17]. Comparative experiments are conducted on three maps of sizes 20 × 20, 40 × 40, and 100 × 100. The planning time for each experiment is taken as the average of 50 repeated experiments. For sampling-based algorithms, the search step size is uniformly set to 1.4 grid cells, and the number of iterations is set to 500.

#### 4.1.1. The 20 × 20 Environment Experimental Analysis

**Figure 12:**
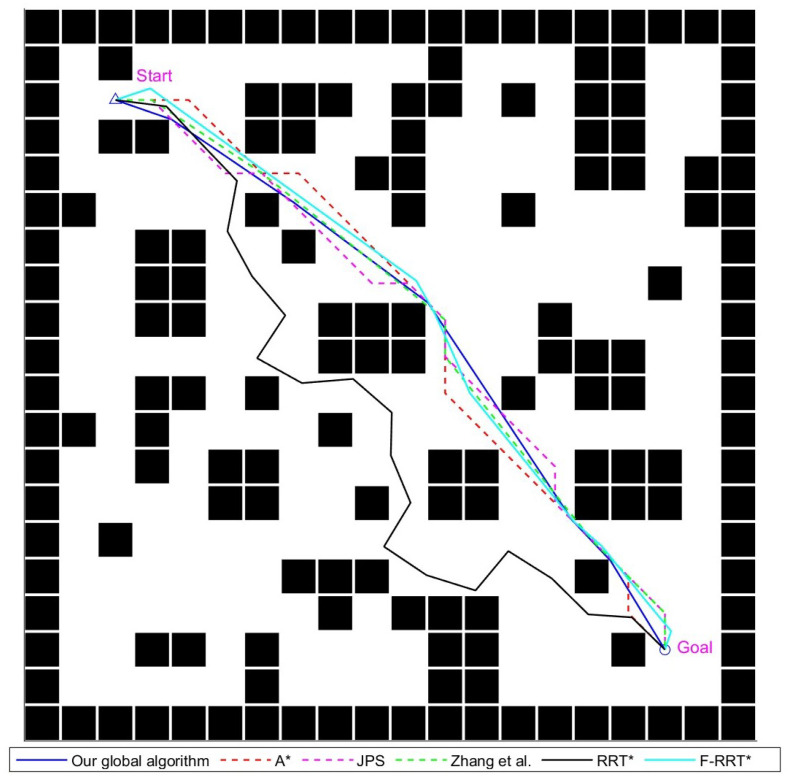
Various global path-planning algorithms diagram for the 20 × 20 map environment, Zhang et al. proposed the improved JPS algorithm in reference [22], written by Zhang et al. in 2021.

This experiment illustrates the performance of various path-planning algorithms in a small-scale scenario. The experimental scene and planned paths are shown in Figure 12, the experimental results are shown in Table 2. The algorithm from reference [22] reduces the path length by 3.2% compared to classic algorithms due to its strategy for eliminating redundant path points. It also achieves an average reduction of 56.8% in the number of path nodes and 53.0% in total path angles. However, due to dense obstacles and the optimization relying solely on the reduction of existing path points, the optimized route still has some redundancy.

The two sampling-based algorithms complete path searching through random exploration and iterative optimization. In terms of the number of path turns, the F-RRT* algorithm successfully reduces the number of turns and complexity by improving the parent node selection mechanism and eliminating unnecessary intermediate nodes. However, its search speed is constrained by the iterative optimization process, making its overall efficiency inferior to other graph-based search algorithms.

The improved algorithm proposed in this paper is significantly more efficient than the A* algorithm. By using a binary search approach to further refine the path and employing bidirectional optimization, this method outperforms both classic algorithms and the reference algorithm in terms of path length, node count, and angles. Compared to classic graph search-based algorithms, it reduces the path length by an average of 5.5%, the number of nodes by 62.1%, and the total turning angle by 84.5%. Compared to the reference algorithm, it shows more pronounced optimization effects and better path quality.

#### 4.1.2. The 40 × 40 Environment Experimental Analysis

**Figure 13:**
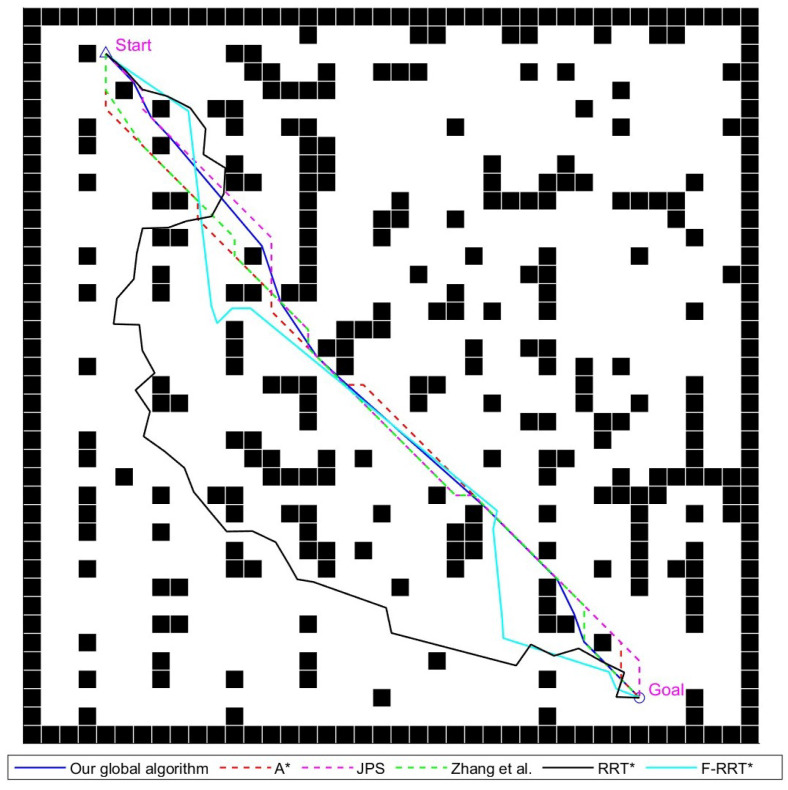
Various global path-planning algorithms diagram for the 40 × 40 map environment, Zhang et al. proposed the improved JPS algorithm in reference [22], written by Zhang et al. in 2021.

This experiment illustrates the performance of various path-planning algorithms in a medium-sized scenario. The experimental scene and planned paths are shown in Figure 13, the experimental results are shown in Table 3. As the map size increased, the efficiency advantage of the JPS algorithm, which uses a jump point search strategy, became more apparent. Its processing time was only 43.0% of that of the A* algorithm. With a larger number of nodes to handle, the jump point search strategy retains only the necessary nodes, resulting in a significantly lower number of nodes compared to the A* algorithm.

The RRT* algorithm exhibits detours because of its randomness, while the F-RRT* algorithm avoids excessive redundant paths due to path optimization strategies similar to those in reference [22] and this study.

The algorithm from reference [22] reduces the number of path nodes by an average of 65.7% compared to classic graph search-based algorithms. However, it does not change the total number of path angles, and the path length is only shortened by 0.4%. In the experimental environment, many obstacles are located near path points. Frequent collisions with obstacles during path point reduction leads to less noticeable optimization effects.

In contrast, the algorithm proposed in this paper reduced the path length by 3.2%, the number of nodes by an average of 61.7%, and the total turning angle by an average of 64.6%. Overall, our algorithm remains significantly better than the existing global path-planning algorithms.

#### 4.1.3. The 100 × 100 Environment Experimental Analysis

**Figure 14:**
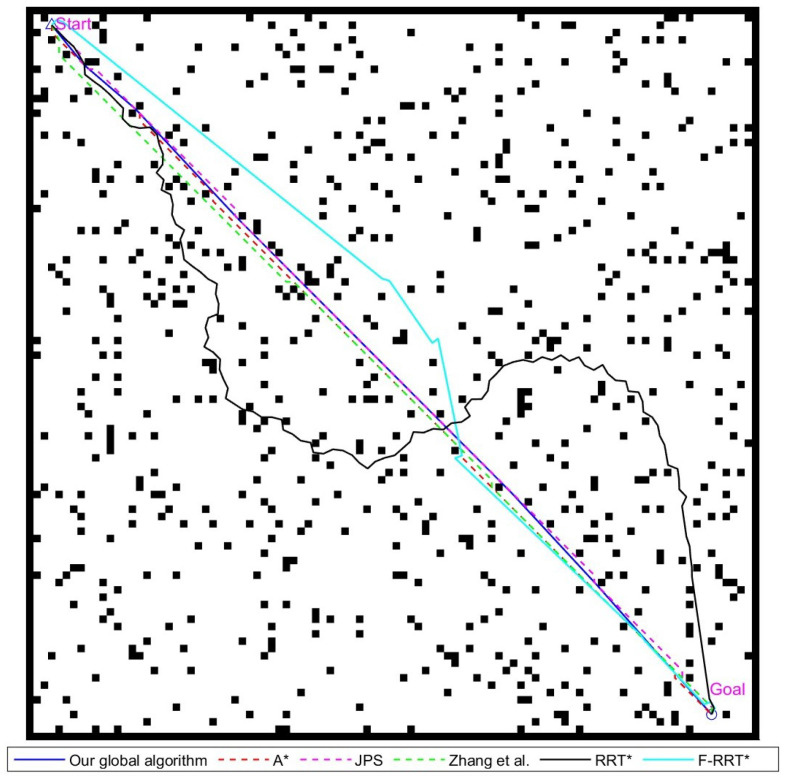
Various global path-planning algorithms diagram for the 100 × 100 map environment, Zhang et al. proposed the improved JPS algorithm in reference [22], written by Zhang et al. in 2021.

This experiment demonstrates the performance of various path-planning algorithms in a large-scale scenario. The experimental scene and planned paths are shown in Figure 14, the experimental results are shown in Table 4. In this scenario, the density of obstacles is lower compared to the previous two scenarios, but the total number of obstacles significantly increases. As a result, frequent encounters with obstacles and the lack of effective path optimization strategies lead to a substantial increase in the number of path nodes for both A* and JPS. For the A* algorithm, the increased map size further widens the gap in search time compared to other graph-based search algorithms.

The algorithm from reference [22] significantly reduced the number of path nodes due to its mechanism for eliminating redundant path points. However, since it cannot adjust the positions of existing path points, the resulting path still has relatively sharp turns. The RRT* algorithm, heavily influenced by randomness as the map size increases, exhibited more significant detours. While the F-RRT* algorithm mitigated detours with its path point reconstruction mechanism, it still demonstrated some redundancy in paths.

In contrast, our global algorithm maintained superior path quality without a notable increase in search time. Overall, our global path-planning algorithm proposed in this paper outperformed other existing algorithms in the large-scale scenario.

### 4.2. Comparative Experiment Analysis of Fusion Path-Planning Algorithms

In this section, comparative experiments are conducted using the improved fusion algorithm from this paper and existing algorithms across various general static scenes, including medium-sized and large-scale environments.

In addition, to independently verify the optimization effect of the local path-planning algorithm, controlled variable comparison experiments are conducted in this section. These experiments are performed in the static scene with unknown obstacles and in the dynamic scene.

To further reduce collision risk, the experiments in this section employ the inflation radius strategy.

#### 4.2.1. Comparative Experiment Analysis of General Static Scenes

This section analyzes the differences in performance between the improved fusion path-planning algorithm and existing fusion algorithms in general static environments.

**Figure 15:**
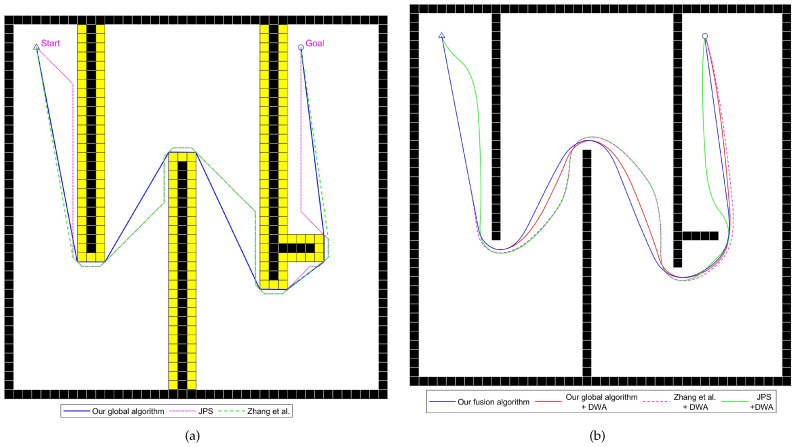
Diagrams of an experiment for static scene 1, with yellow cells representing the inflated space, Zhang et al. proposed the improved JPS algorithm in reference [22], written by Zhang et al. in 2021. (**a**) Paths generated by various global path-planning algorithms. (**b**) Paths traveled by the mobile robot using various fusion path-planning algorithms.

**Figure 16:**
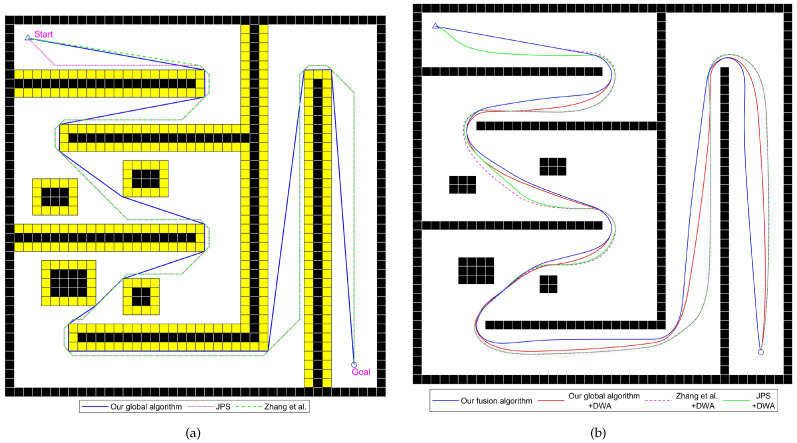
Diagrams of the experiment for static scene 2, with yellow cells representing the inflated space, Zhang et al. proposed the improved JPS algorithm in reference [22], written by Zhang et al. in 2021. (**a**) Paths generated by various global path-planning algorithms. (**b**) Paths traveled by the mobile robot using various fusion path-planning algorithms.

**Figure 17:**
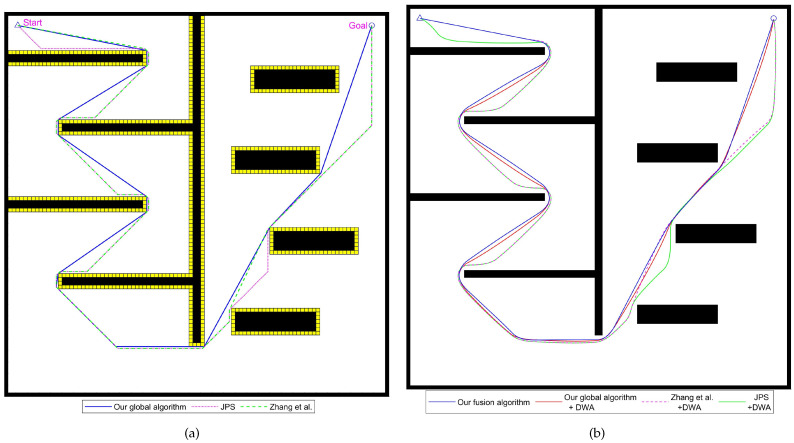
Diagrams of the experiment for static scene 3, with yellow cells representing the inflated space, Zhang et al. proposed the improved JPS algorithm in reference [22], written by Zhang et al. in 2021. (**a**) Paths generated by various global path-planning algorithms. (**b**) Paths traveled by the mobile robot using various fusion path-planning algorithms.

The experimental scenes and planned paths are shown in Figure 15, Figure 16 and Figure 17, and the experimental results are listed in Table 5, Table 6 and Table 7. Static scene 1 involves multiple walls blocking the path, requiring the device to frequently turn to reach the target. Static scene 2 contains multiple walls and some obstacles of considerable size. Static scene 3 features a larger size and is used to showcase the performance of various integrated algorithms in large-scale scenarios.

In static scene 1, the path planned by the improved global path-planning algorithm presented in this paper is 6.7% shorter than the JPS algorithm and 4.1% shorter than the algorithm from reference [22], with the total turning angle reduced by 44.3% and 20.9%, respectively. Although the algorithm from reference [22] improves path quality to some extent, the presence of obstacles blocking certain corners prevents full optimization. For the two algorithms using the improved global path-planning algorithm, the travel length and time are significantly shorter than those of the other two algorithms, with the travel length reduced by an average of 4.3% and the travel time reduced by an average of 4.7%. The improved fusion path-planning algorithm, with paths more closely following the initially planned global path, further reduces travel length and time, with the travel length reduced by 0.2% and travel time by 1.0% compared to the DWA algorithm fused with the improved global algorithm. The average linear velocity also shows some improvement due to dynamic parameter adjustment, while the average angular velocity decreases by 3.3%, indicating that the improved local path algorithm can reduce turning operations to some extent, enhancing the stability of the device during operation.

In static scene 2, the path planned by the improved global path-planning algorithm proposed in this paper is 5.0% shorter than the JPS algorithm and 4.4% shorter than the algorithm from reference [22], with path angles reduced by 21.2% and 17.2%, respectively, as shown in Figure 16. Due to frequent obstructions by obstacles and walls, the optimization effect of the algorithm from reference [22] is very limited, and it can only remove redundant paths before bypassing the first wall, offering no advantage in travel distance. For the fusion algorithms using the improved global planning algorithm, travel length and time are significantly reduced, with the travel length reduced by an average of 4.8% and travel time by 5.3%. The improved fusion path-planning algorithm, compared to the DWA fused with the improved local algorithm, achieves a 0.96% reduction in travel length and a 1.4% reduction in travel time, resulting in better travel efficiency.

Static scene 3 simulates the performance of various algorithms on a large-scale map. On the left side of the scene, multiple walls are present to test the continuous turning performance of integrated algorithms, while the right side features large obstacles to evaluate the algorithms’ avoidance capabilities in scenarios with multiple obstacles. As shown in Figure 17b, the proposed algorithm demonstrates fewer unnecessary turning operations and generates relatively smoother paths compared to other algorithms. According to the data in Table 7, the proposed algorithm achieves shorter paths and fewer turning operations and allows the mobile robot to travel smoothly at higher speeds. This results in greater efficiency and stability in large-scale map scenarios.

#### 4.2.2. Comparative Experiment Analysis of the Static Scene with Unknown Obstacles

**Figure 18:**
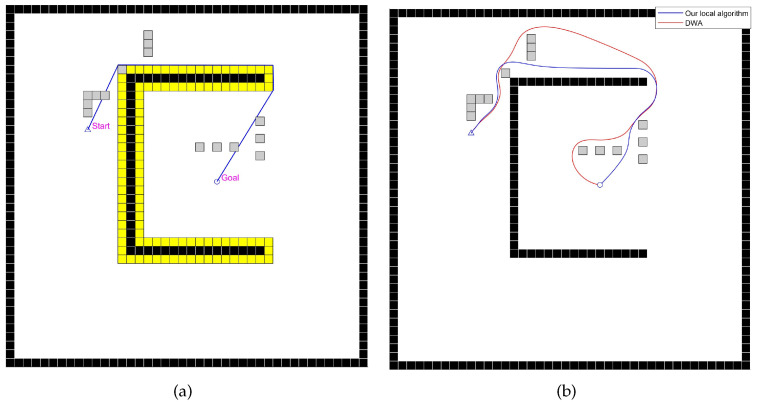
Diagrams of the experiment for the static scene with unknown obstacles, with gray cells representing the unknown space. (**a**) The blue path is the route planned by our improved global path-planning algorithm, with yellow cells representing the inflated space. (**b**) Paths planned by the mobile robot using the DWA algorithm and our improved local path-planning algorithm.

This section analyzes the differences in performance between the improved local path-planning algorithm and the existing algorithm in a static environment with unknown obstacles. The global path-planning algorithm uniformly uses the improved global path-planning algorithm proposed in this paper to control variables. The experimental scenario and planned paths are shown in Figure 18, and the results are presented in Table 8.

This scene features a U-shaped wall and unknown obstacles. As shown in Figure 18, the improved local path-planning algorithm includes a new evaluation factor p(v,w). This allows the robot to quickly return to the planned global path after temporarily deviating to avoid unknown obstacles, thus reducing travel length and time. The DWA algorithm, however, only guides the robot to the next target point, resulting in several unnecessary detours in this experiment and extending the travel time. In this experiment, the improved local path-planning algorithm reduced travel length and time by 17.9% compared to the DWA algorithm, and it also decreased the average angular velocity by 10.9%. Overall, the improved local path-planning algorithm is clearly superior to the DWA algorithm.

#### 4.2.3. Comparative Experiment Analysis of the Dynamic Scene

**Figure 19:**
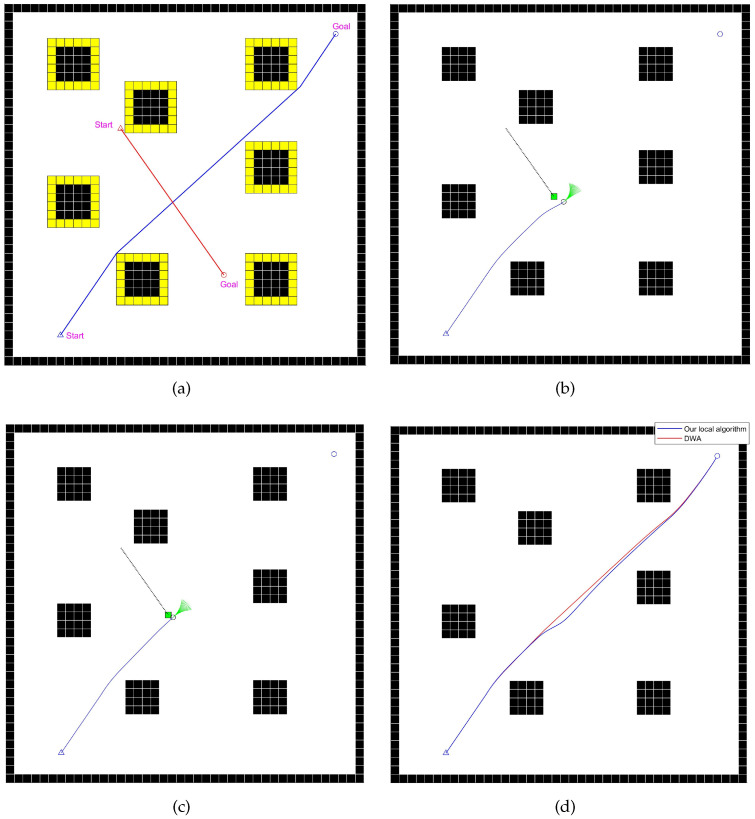
Diagrams of the experiment for dynamic scene. (**a**) The blue and red paths respectively represent the planned paths of the mobile robot and the dynamic obstacle, with yellow cells representing the inflated space. (**b**) Diagram of the moment when the device using our improved local path-planning algorithm is closest to the dynamic obstacle, where the green rectangle represents the dynamic obstacle, and the black circle represents the mobile robot. The blue line represents the route that the robot has already traveled. (**c**) Diagram of the moment when the device using the DWA algorithm is closest to the dynamic obstacle. The blue line represents the route that the robot has already traveled. (**d**) Travel paths of the two fusion algorithms.

This section verifies the effectiveness of the improved local path-planning algorithm in a dynamic scenario with dynamic obstacles. The global path-planning algorithm used is the improved version proposed in this paper. The experimental scenario and planned paths are shown in Figure 19, and the results are presented in Table 9.

In this scene, there are a few static and dynamic obstacles, with the paths of the mobile robot and the dynamic obstacles intersecting, posing a risk of lateral collision. As shown in Figure 19b, the improved local path-planning algorithm, due to the presence of the dyn(v,w) evaluation factor, allows the device to actively choose a speed space with the lowest collision risk when approaching dynamic obstacles. In contrast, Figure 19c shows that the device using the DWA algorithm relies only on the dist(v,w) scoring factor to avoid dynamic obstacles, leading to insufficient avoidance and, ultimately, a collision with the dynamic obstacle.

In this experiment, the minimum distance between the mobile robot and the dynamic obstacle during its movement improved by 104.8% with the improved local path-planning algorithm compared to the DWA algorithm, significantly enhancing the safety of the device during operation without noticeably reducing its travel efficiency. Therefore, it performs better in the dynamic scene.

## 5. Conclusions and Future Work

This paper addresses the issues of path redundancy and excessive turning points in global path-planning algorithms by improving the heuristic function’s search strategy and the path generation method, and it also resolves problems in local path-planning algorithms, such as their tendency to fall into local optima and their failure to avoid dynamic obstacles in time, by introducing a dynamic parameter adjustment strategy and adding evaluation factors for deviation and dynamic obstacle avoidance. Finally, the fusion of these two improved algorithms, along with the introduction of an inflation radius to further prevent collisions between the device and obstacles, forms the improved fusion path-planning algorithm. This algorithm enhances the adaptability of mobile robots in various environments. Simulation experiments were conducted using the improved path-planning algorithm in standard environments, environments with unknown obstacles, and dynamic environments. The experiments demonstrate that, compared to existing algorithms, the improved fusion path-planning algorithm results in shorter travel distances, greater stability in static environments, and higher safety in dynamic environments, providing significant advantages. Overall, the improved fusion path-planning algorithm outperforms existing algorithms across various scenarios.

In future work, we plan to enhance the resolution of the grid map and refine the areas occupied by obstacles, thereby reducing the proportion of occupied areas in the entire grid map. This will minimize the impact of inflated space on the mobile robot’s movement on the map and alleviate the problem of paths being blocked by inflated space. Additionally, we aim to further improve our local path-planning algorithm’s real-time adaptability in highly dynamic environments by optimizing the evaluation factors of the local path-planning algorithm; it will adopt more diverse obstacle avoidance strategies, such as following or stopping and waiting, based on the specific situation of dynamic obstacles, thereby further enhancing safety and flexibility.

## Figures and Tables

**Table 1:** The relationship between $\theta_{pos}$ and $\theta_{vel}$ in various collision risk scenes.

Diagram	$\theta_{\mathrm{pos}}$	$\theta_{\mathrm{vel}}$	Collision Risk Level
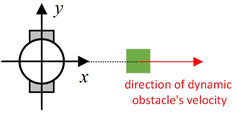	0°	0°	1
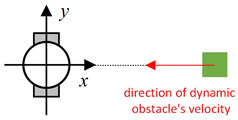	0°	180°	3
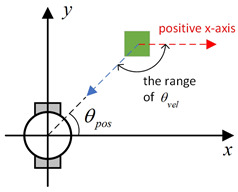	(0°, 90°)	(180° + $\theta_{pos}$,360°)	2
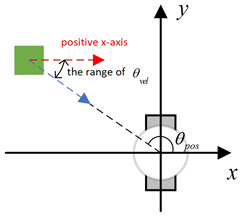	[90°, 180°)	(180° + $\theta_{pos}$,360°)	1
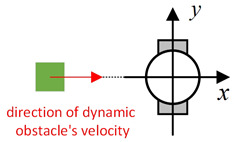	180°	0°	1
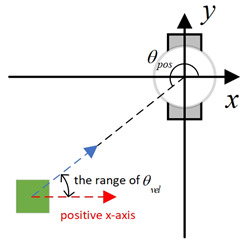	(180°, 270°)	(0°,$\theta_{pos}-$ 180°)	1
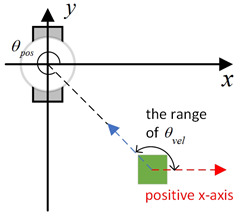	[270°, 360°)	(0°,$\theta_{pos}-$ 180°)	2

**Table 2:** Experimental results for global path-planning algorithms in the 20 × 20 map environment; the best results are highlighted.

Global Algorithm	Calculation Time (ms)	Path Length	Number of Nodes	Total Turning Angle (°)
A*	32	22.97	19	315
JPS	**22**	22.97	18	450
Zhang et al. [22]	23	22.24	8	180
RRT*	44	27.4	21	868°
F-RRT*	84	22.41	12	510°
Our global algorithm	23	**21.71**	**7**	**59.38**

**Table 3:** Experimental results for global path-planning algorithms in the 40 × 40 map environment; the best results are highlighted.

Global Algorithm	Calculation Time (ms)	Path Length	Number of Nodes	Total Turning Angle (°)
A*	79	47.59	37	405°
JPS	**34**	47.59	31	405°
Zhang et al. [22]	35	47.37	12	405°
RRT*	315	65.71	49	1753°
F-RRT*	723	51.9	14	510°
Our global algorithm	35	**46.09**	**12**	**143.26°**

**Table 4:** Experimental results for global path-planning algorithms in the 100 × 100 map environment; the best results are highlighted.

Global Algorithm	Calculation Time (ms)	Path Length	Number of Nodes	Total Turning Angle (°)
A*	121	131.86	96	495°
JPS	**44**	131.86	83	495°
Zhang et al. [22]	45	131.68	9	296.57°
RRT*	2180	185.73	134	4113.69°
F-RRT*	3260	138.19	15	778.59°
Our global algorithm	48	**130.23**	**7**	**21.89°**

**Table 5:** Comparison of experimental results for static scene 1; the best results are highlighted.

Fusion Algorithm	Global Path Length	Global Path Turning Angle (°)	Robot Travel Length	Robot Travel Time (s)	Average Linear Velocity	Average Angular Velocity
JPS + DWA	98.59	765	94.86	162.79	0.582	4.45
Zhang et al. [22] + DWA	96.00	538.26	94.25	161.49	0.583	3.42
Our global algorithm + DWA	92.01	425.57	90.57	155.29	0.583	3.03
Our fusion algorithm	**92.01**	**425.57**	**90.34**	**153.67 **	**0.587**	**2.93**

**Table 6:** Comparison of experimental results for static scene 2; the best results are highlighted.

Fusion Algorithm	Global Path Length	Global Path Turning Angle (°)	Robot Travel Length	Robot Travel Time (s)	Average Linear Velocity	Average Angular Velocity
JPS + DWA	181.49	1125	176.58	297.08	0.594	3.79
Zhang et al. [22] + DWA	180.49	1071.03	176.88	296.28	0.597	3.5
Our global algorithm + DWA	172.4	886.29	169.13	282.98	0.597	3.33
Our fusion algorithm	**172.4**	**886.29**	**167.5**	**278.96**	**0.600**	**3.32**

**Table 7:** Comparison of experimental results for static scene 3; the best results are highlighted.

Fusion Algorithm	Global Path Length	Global Path Turning Angle (°)	Robot Travel Length	Robot Travel Time (s)	Average Linear Velocity	Average Angular Velocity
JPS + DWA	288.04	1035	283.34	443.56	0.606	2.35
Zhang et al. [22] + DWA	284.22	889.7	280.75	462.87	0.606	1.94
Our global algorithm + DWA	272.91	606.9	270.61	445.27	0.607	1.53
Our fusion algorithm	**272.91**	**606.9**	**270.45**	**443.57**	**0.610**	**1.45**

**Table 8:** Comparison of experimental results for the static scene with unknown obstacles; the best results are highlighted.

Local Algorithm	Robot Travel Length	Robot Travel Time (s)	Average Linear Velocity	Average Angular Velocity
DWA	51.31	92.82	0.552	6.46
Our local algorithm	**42.12**	**76.21**	**0.552**	**5.75**

**Table 9:** Comparison of experimental results for the dynamic scene, where “Closest distance” refers to the minimum distance between the robot and the dynamic obstacle during its journey.

Local Algorithm	Robot Travel Length	Robot Travel Time (s)	Average Linear Velocity	Average Angular Velocity	Closest Distance
DWA	**47.66**	84.51	0.566	**0.457**	0.62
Our local algorithm	47.75	**84.08**	**0.567**	0.968	**1.27**

## Data Availability

Data are contained within the article.
